# Short-term results in canines of novel stent-graft design for chimney technique in TEVAR

**DOI:** 10.1016/j.jimed.2020.07.004

**Published:** 2020-07-09

**Authors:** Ming Li, Chang Shu, Benhao Xiao, Dingxiao Liu, Weichang Zhang

**Affiliations:** a Department of Vascular Surgery, The Second Xiangya Hospital, Central South University, Changsha, 410011, China; b Department of Vascular Surgery, Fuwai Hospital, Chinese Academy of Medical Sciences, Beijing, 100037, China; c Lifetech Scientific Co. Ltd, China

**Keywords:** Thoracic endovascular aortic repair, Chimney technique, Canine model, In vivo experiment, Stent-graft

## Abstract

**Introduction:**

Parallel stent-stent grafting is a major endovascular technique used to preserve the supra-aortic branches during thoracic endovascular aortic repair (TEVAR) of aortic pathologies involving the aortic arch. The short- and mid-term results of this technique are satisfactory; however, endoleak remains a major concern. Thus, here we designed a new chimney stent-graft to decrease the endoleak rate.

**Aim:**

To testify the feasibility and safety of the new chimney stent-graft system in a canine model.

**Material and methods:**

Six Labrador retrievers were used. Pre-operative data were collected and all operations were performed under general anesthesia. The main and chimney stent-grafts were implanted through the abdominal aorta and left subclavian artery approaches, respectively. Completion digital subtraction angiography (DSA) was performed to confirm the immediate outcomes. All dogs were fed separately for 6 months and sacrificed after aortic angiography. The thoracic aorta and the main and chimney stent-grafts were harvested for histopathologic examination.

**Results:**

No complications were found in follow-up DSA. All branch arteries were patent. Inflammatory responses were observed around the stent-grafts in 3 experimental animals, and slight hyperplasia was observed in the surrounding tissues compared with the normal vessels. There was no mural thrombus in the stent, endothelial cells were noted on the inner surface of the stent, and thrombus was formed in the outer skirt and gutter area. The histopathologic examinations revealed similar results to those of gross necropsy observations.

**Conclusions:**

This study demonstrated the feasibility and safety of the Longuette stent-graft and the first to report a revised stent-graft specific for chimney technique.

## Introduction

Chimney and fenestration/branch techniques are the most commonly used endovascular techniques to preserve the supra-arch branches in thoracic endovascular aortic repair (TEVAR). Although satisfactory results were reported in various centers, endoleak remains a major concern. Type I endoleak occurs in 7–23.1% of cases treated using the chimney technique, mostly from the gutter between the chimney and aortic stents.^1^, ^2^, ^3^, ^4^, ^5^, ^6^, ^7^, ^8^ Here we designed a new chimney stent-graft aiming to decrease the volume of the gutter area and thereby reduce the endoleak rate and tested its feasibility and safety in a canine model.

## Material and Methods

### Ethical approval

The study was approved by the ethics committee of the Second Xiangya Hospital.

### Device description

**Fig. 1:**
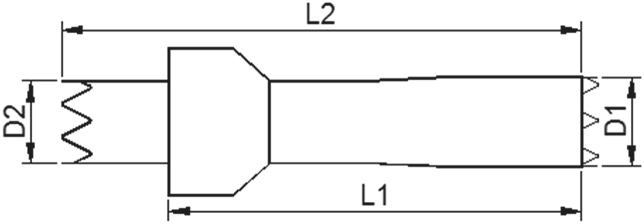
The scheme of stent-graft design.

The self-expanding stent-graft is composed of a Ni-Ti metal scaffold and an expanded polytetrafluoroethylene graft woven to the surface of the scaffold with a “skirt-shaped” structure. After its release, the outer stent-graft fills the gutter area outside the inner main chimney stent aiming to decrease the incidence of endoleak. Additionally, the inner main stent is designed with greater radial force than the outer stent to ensure its patency (Fig. 1). The stent-graft is packed in a 10–12F delivery system according to its diameter and is released in a self-expandable manner. It is given the name “Longuette” because of its skirt-like shape.

### Implantation

The surgical experiment was performed in 6 Labrador retrievers at the Advanced Animal Study Services Center, Shenzhen, China, to test the safety and feasibility of the device system. The animals were provided by Jiagan Biotech Co. Ltd., Shanghai, China. The mean pre-operative weight was 29.1 ​± ​1.85 ​kg. The pre-operative profiles of the animals are listed in Table 1.

**Table 1:** Pre-operative profiles.

Animal serial number	Sex	Implant weight (kg)	Study duration (months)
A629	F	31.0	6
A710	F	30.0	6
A711	M	25.7	6
A798	M	30.0	6
A901	F	28.5	6
A903	F	29.1	6

The anatomic data were acquired by pre-operative digital subtraction angiography (DSA) in all animals 7 days prior to surgery. The aortic and chimney stent-grafts were prepared based on the DSA measurements. All the procedures were performed under general anesthesia.

**Fig. 2:**
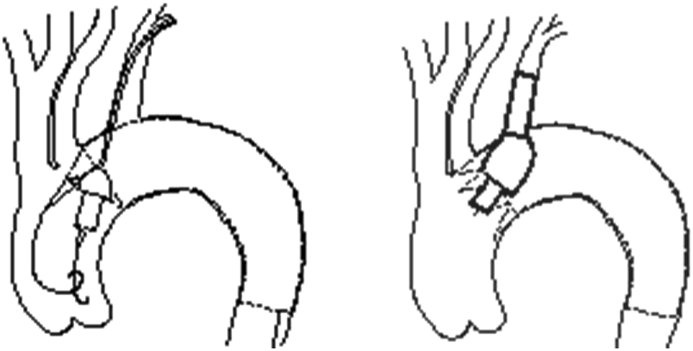
Schematic diagram of the deployment of chimney stent-graft.

After intramuscular premedication with zolezapam (5 ​mg/kg body weight [BW]), intravenous anesthesia was induced by propofol (8 ​mg/kg BW) and maintained by the continuous inhalation of isoflurane. The dogs were endotracheally intubated and ventilated at 200 ​mL ​at 10–16 breaths per minute using 30% oxygen. Heparin (300 IU/kg BW) was given intravenously for anticoagulation. A mid-abdominal incision was made and the abdominal aorta was carefully dissected. A 6F sheath was introduced into the abdominal aorta. Another incision was made in the left neck and the left subclavian artery (LSA) was dissected. A 5F sheath was introduced into the LSA. The pigtail catheter was advanced into the ascending aorta over a 0.035” guidewire. Then the guidewire was removed, and angiography was performed via the pigtail catheter. The diameters of the aortic arch and the LSA were remeasured for final confirmation of the stent-graft choices. Lunderquist guidewires were then advanced to the ascending aorta via the abdominal and LSA access points, respectively. Both sheaths were removed and delivery systems of both stent-grafts were advanced through the abdominal aorta and the LSA access points, respectively. The aorta stent-graft was first deployed at the aortic arch distal to the orifice of the innominate artery with total coverage of the LSA. After the deployment of the aorta stent-graft, the chimney stent-graft was half released until the outer layer was fully expanded. The entire delivery system was then carefully retracted until the outer layer was close and proximal to the orifice of the LSA (Fig. 2). Completion DSA was then performed to confirm the immediate outcome. All incisions were closed after retraction of the guidewires and catheters.

After recovering from anesthesia, the dogs were fed separately and continuously observed for appetite, movement, and mental state. Dual antiplatelet therapy consisting of clopidogrel 75 ​mg and aspirin 100 ​mg per day was given to all dogs to prevent thrombosis. Cefotaxime sodium was administered for 3 consecutive days postoperatively in all dogs. All dogs underwent a second aortic angiography to exclude adverse events and were sacrificed 6 months after stent-graft implantation by the intravenous injection of 10% potassium chloride. The entire thoracic aorta and stent-grafts were acquired for gross pathology and histological evaluations.

## Results

Stent-graft deployment was successful in all six dogs. No major bleeding or cardiac events occurred throughout the preparation and implantation periods. No retrograde type A dissections occurred during implantation.

The anatomic data were acquired by pre-operative DSA. The anatomic features and stent-graft types are listed in Table 2.

**Table 2:** Aortic anatomy and stent-graft data.

Serial number	Aortic arch type	Distance between supra-arch branches (mm)	Implantation position	Stent-graft diameter (mm)	Aorta diameter (mm)			Average aorta diameter (mm)
					P	M	D	
A629	I	5	Aorta	22	21.8	22	21.7	21.83
			Branch	10	9.4	9.3	9.7	9.47
A710	III	6	Aorta	24	24.0	23.1	23.4	23.50
			Branch	10	9.5	9.4	9.7	9.53
A711	II	5	Aorta	22	21.4	21.0	21.8	21.40
			Branch	10	9.6	9.2	9.7	9.50
A798	I	3	Aorta	22	21.4	21.0	21.8	21.40
			Branch	8	7.9	7.2	7.6	7.57
A901	II	6	Aorta	22	21.5	21.1	21.9	21.50
			Branch	10	9.5	9.1	9.8	9.47
A903	I	3	Aorta	22	21.6	21.1	21.3	21.33
			Branch	8	7.9	7.5	7.7	7.70

P: Proximal landing zone; M: Middle part of target vessel; D: Distal landing zone.

**Fig. 3:**
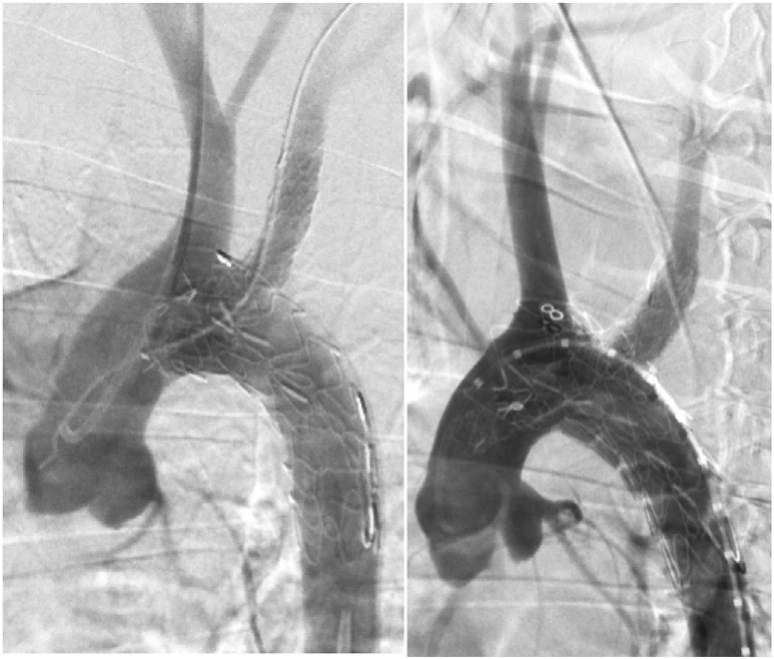
Examples of completion DSA.

The mean procedure time was 149 ​± ​16.6 ​min. The abdominal aorta and LSA were exposed safely without complications. The mean procedure time for exposure was 20 ​± ​2.94 ​min. Stent-graft delivery and deployment occurred precisely without accident. On the completion DSA, the stent-graft positions were confirmed to be fixed without migration. All branch arteries were patent (Fig. 3).

**Fig. 4:**
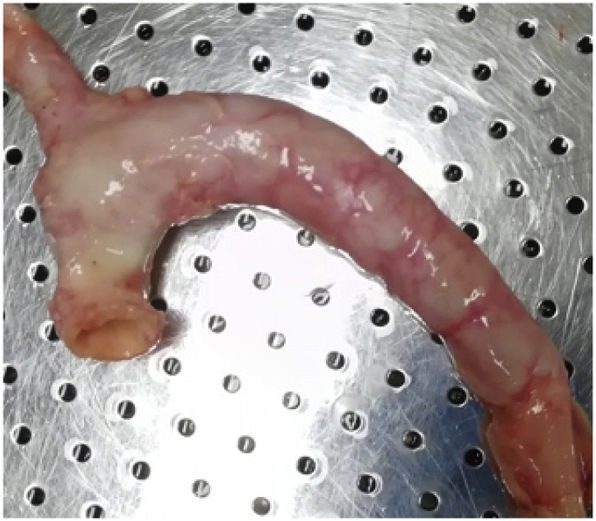
The whole thoracic aorta was harvested.

Postoperative appetite, mobility, and mental status were normal in all dogs. Mean post-operative weight was 29.3 ​± ​1.95 ​kg. No complications were found prior to sacrifice. After follow-up DSA was performed at 6 months, all dogs were sacrificed under general anesthesia and the thoracic aorta was harvested and embedded in paraffin for further histological staining and analysis (Fig. 4).

**Fig. 5:**
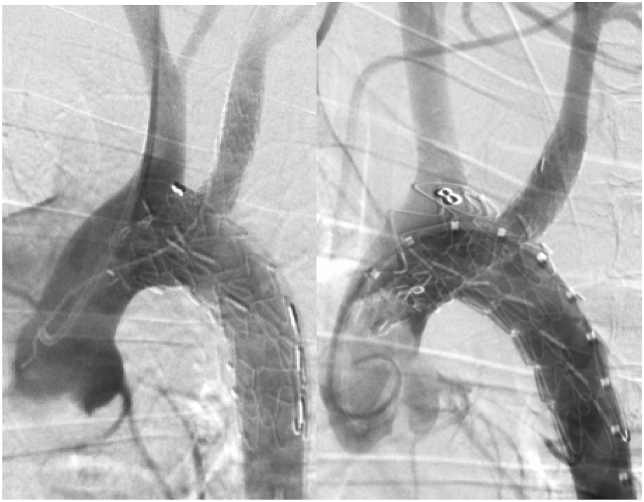
Follow-up DSA at 6 months.

No complications were found on the follow-up DSA. All branch arteries were patent (Fig. 5). No dissection or hematoma was found on the gross necropsy. Inflammatory responses were observed around the stent-grafts in 3 experimental animals, and slight hyperplasia was observed in the surrounding tissues compared with the normal vessels. There was no mural thrombus in the stent, endothelial cells were noted on the inner surface of the stent, and thrombus was noted in the outer skirt and gutter area.

**Fig. 6:**
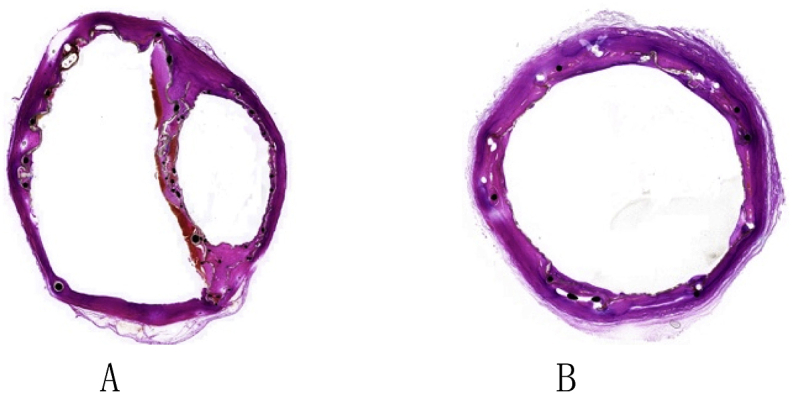
Histopathology results of thoracic aorta. A: In the gutter area; B: Vessel distal to chimney stent-graft.

The histopathologic exams showed similar results to those of the gross necropsy observations. Slight inflammation was observed in the vessels at the implantation site. A small number of inflammatory cells were visible in the tissue surrounding the scaffold and the coated material, but no mural thrombi were noted in the main and chimney stent-grafts (Fig. 6).

## Discussion

The main technical problem with the chimney technique is the risk of type Ia endoleak through the gutters between the main aortic and the parallel graft.^1^ The endoleak rate after the chimney technique in TEVAR is reportedly 7–23.1%.^1^, ^2^, ^3^, ^4^, ^5^, ^6^, ^7^, ^8^ Because the chimney technique involves the placement of multiple stent-grafts, endoleaks commonly occur from the gutters between devices.^7^ This revised stent-graft was designed to eliminate gutter flow by the addition of an “outer graft” to a normal stent-graft. It was given the name “Longuette” because of its shape. This experimental study showed that the “outer skirt” effectively blocked blood flow from the gutter area.

Although there is insufficient evidence regarding the performance of bare and covered stents as chimney stents, some experts believe that the risk of perfusion into the gutters and retrograde flow from the aortic arch is greater with bare than covered stents.^6^^,^^9^ Our clinical experience with over 200 cases of chimney TEVAR suggests that the covered stent is the better choice. A covered stent can prevent endoleak through the mesh of bare stents, while the outer skirt of the Longuette stent-graft can further block proximal endoleaks and facilitate thrombosis formation in the gutter area, thus decreasing the type Ia endoleak rate after chimney TEVAR. Because of its unique design, it is unlikely to be deployed in a balloon-expandable manner.

The possibility of the “outer skirt” unintentionally covering the adjacent branch artery may be worrisome. In this experimental study, no such complications were found. The “outer skirt” is 6–10 ​mm larger than the main graft; depending on the diameter, it is unlikely to cover the adjacent artery. Moreover, the Longuette stent-graft can be deployed more proximally into the aorta cavity if the orifices of the branch arteries are very close together.

Finally, the experimental study has limitations. Besides the limited number of animals and short observation time, further studies should be performed in pathological models like aneurysm or dissection. The possible interference between multiple Longuette stent-grafts should also be tested.

## Conclusion

The Longuette stent-graft is the first reported revised stent-graft specific for chimney technique. This experimental study proved the feasibility and safety of this stent-graft. These results suggest that the use of this graft might improve clinical outcomes after chimney TEVAR.

## Declaration of competing interest

The authors declare that they have no conflicts of interests to this work. We declare that we do not have any commercial or associative interest that represents a conflict of interest in connection with the work submitted.
